# Preferable background filtering for next-generation sequencing analysis in non-small cell lung cancer: pericarcinomatous tissues or peripheral blood lymphocytes?

**DOI:** 10.1186/s40880-019-0378-4

**Published:** 2019-06-13

**Authors:** Yaxiong Zhang, Lianpeng Chang, Wenfeng Fang, Yunpeng Yang, Lanjun Zhang, Shaodong Hong, Huaqiang Zhou, Yanfang Guan, Xin Yi, Li Zhang

**Affiliations:** 1 State Key Laboratory of Oncology in South China, Collaborative Innovation Center for Cancer Medicine, Sun Yat-sen University Cancer Center, Guangzhou, 510060 Guangdong P. R. China; 2 Department of Medical Oncology, Sun Yat-sen University Cancer Center, 651 Dongfeng Road East, Guangzhou, 510060 Guangdong P. R. China; 3 Geneplus-Beijing Institute, Beijing, 100000 P. R. China; 4 Department of Thoracic Surgery, Sun Yat-sen University Cancer Center, Guangzhou, 510060 Guangdong P. R. China

Dear editor,

Lung cancer is the leading cause of cancer-related death worldwide, with the predominant pathological type being non-small cell lung cancer (NSCLC) [1, 2]. Next-generation sequencing (NGS) analysis is increasingly used to help clinicians select appropriate target therapies, such as epidermal growth factor receptor-tyrosine kinase inhibitors (EGFR-TKIs) for *EGFR*-mutant patients [3]. Both pericarcinomatous tissues and peripheral blood lymphocytes are widely used as normal control for NGS analysis. However, whether pericarcinomatous tissue is suitable for background filtering in mutation analysis remains controversial. According to the whole-genome sequencing data from The Cancer Genome Atlas (TCGA) database, there were some genomic variations in pericarcinomatous tissue from NSCLC patients, but no driver gene mutation was detected [4, 5]. Therefore, deep sequencing of pericarcinomatous and tumor tissues is necessary to confirm whether pericarcinomatous tissue harbors low-frequency mutations.

To determine whether the sequencing data of pericarcinomatous tissues from NSCLC patients can serve as the genomic background in germline mutation profiling, we used a 1021-gene panel (Additional file 1: Table S1) to perform deep targeted capture sequencing of 181 samples of multi-region tumor tissues, 32 samples of matched pericarcinomatous tissues, and 32 samples of matched peripheral blood lymphocytes from 32 patients with resectable NSCLC used as genomic filters. Mutations detected in tumor tissues were defined as tumor-derived mutations. Mutations shared in all multi-region tumor tissues were defined as trunk mutations, and otherwise were branch mutations. Figure 1 shows the process of sample collection. Much more details about methods are shown in Additional file 2: Methods S1. Of the 32 patients with NSCLC, 26 had adenocarcinoma (including 9 with *EGFR* mutation and 6 with kirsten rat sarcoma viral oncogene [*KRAS*] mutation), 5 had squamous cell carcinoma, and 1 had lymphoepithelioma-like carcinoma. The median age at diagnosis was 57 years (range 45–79 years). Most patients were male (78.1%), had stage I–II disease (62.5%), and were smokers (65.6%) (Additional file 1: Table S2). A total of 437 tumor-derived mutations were identified. For tumor tissues, trunk mutations showed a similar proportion (51.7%, 226/437) compared with branch mutations (48.3%, 211/437). For pericarcinomatous tissues, trunk mutations showed a higher proportion (74.1%, 20/27) compared with branch mutations (25.9%, 7/27). Compared with tumor tissues, pericarcinomatous tissues had a significantly higher proportion of trunk mutations (*P *= 0.024). Among the 27 tumor-derived mutations detected in pericarcinomatous tissues, most frequently mutated genes were tumor suppressor genes (TSGs), such as tumor protein 53 (*TP53*). Mutations in either *EGFR* or *KRAS* were not detected in any samples of pericarcinomatous tissues, whereas mutations of some oncogenes, such as v-Raf murine sarcoma viral oncogene homolog B (*BRAF*) and the rearranged during transfection gene (*RET*), were detected in pericarcinomatous tissues (Fig. 2). Six (18.8%) patients had tumor-derived mutations detected in pericarcinomatous tissues. Trunk mutations were detected in 4 patients, whereas branch mutations were detected in 2 patients. Additional file 3: Figure S1 shows the variant allele fraction and number of variant reads of each patient with tumor-derived mutations detected in pericarcinomatous tissues. Furthermore, the detection rate of tumor-derived mutations in pericarcinomatous tissues was irrelevant to age, smoking status, and molecular NSCLC subtype (Additional file 1: Table S2).

**Fig. 1:**
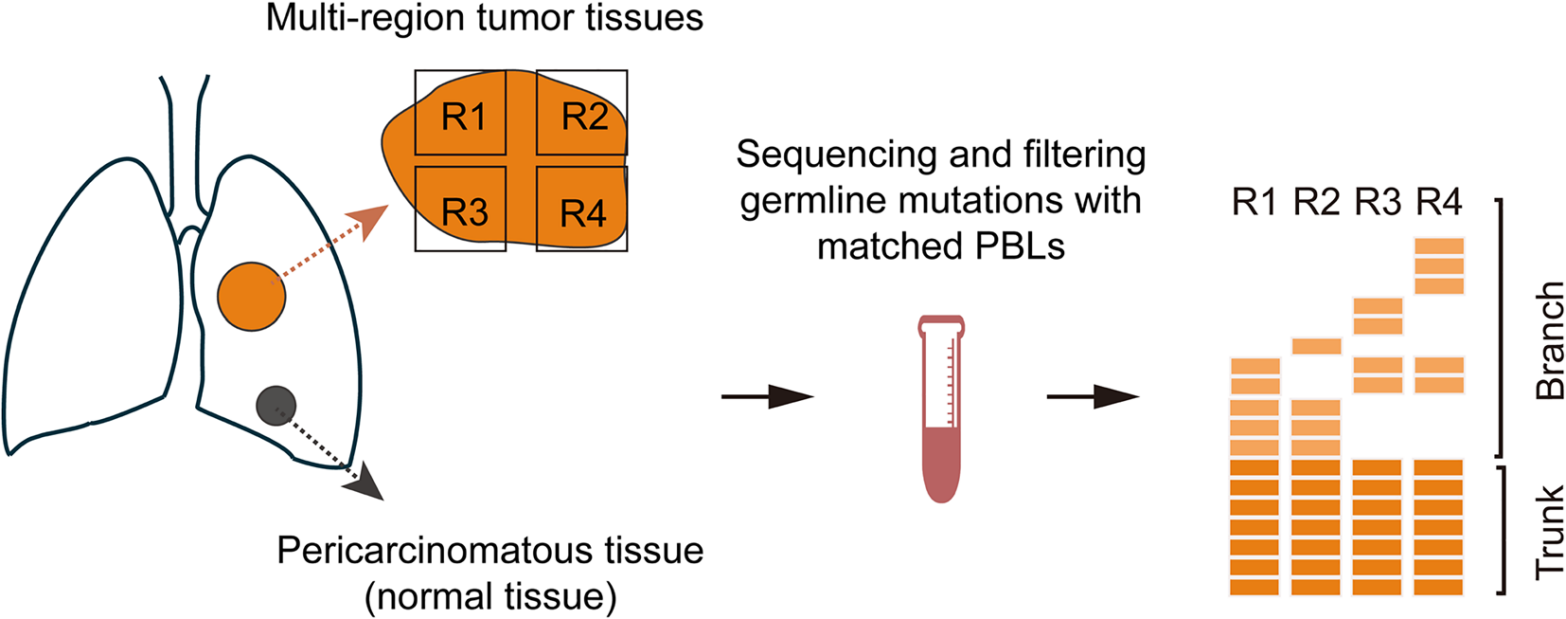
Overview of sample collection and study methodology. Multi-region tumor tissues (e.g., R1–R4) and matched pericarcinomatous tissues (normal tissue) from patients with resectable non-small cell lung cancer (NSCLC) were obtained for deep next-generation sequencing (NGS) using a pan-cancer 1021-gene panel. Matched peripheral blood leucocytes were used as genomic filters. If the somatic genetic alteration was detected in all tissue regions, it was defined as trunk mutation. Otherwise, it was called branch mutation

**Fig. 2:**
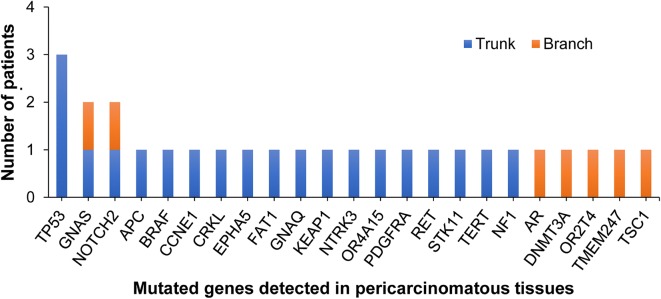
All detected tumor-derived mutations in pericarcinomatous tissues from patients with resectable non-small cell lung cancer (NSCLC)

Genotyping with NGS has been demonstrated to be clinically effective in guiding NSCLC treatment. It helps oncologists to find druggable mutations for targeted therapy [6, 7] and potential targetable genomic alterations for drug development [8, 9]. Previous studies reported the detection of genomic alterations in pericarcinomatous tissues from NSCLC patients [4, 5], but the significance of these mutations in lung carcinogenesis remains unclear. Moreover, it is important to determine whether pericarcinomatous tissue from NSCLC patients can serve as background sample for genomic filtering for germline mutations to increase the accuracy of NGS results.

Thus, we performed deep targeted capture sequencing with pericarcinomatous and tumor tissues. We found that tumor-derived mutations indeed existed in nearly 20% of pericarcinomatous tissues from NSCLC patients, which indicated that pericarcinomatous tissue might not be recommended as background filter for genomic analysis in NSCLC. Neither *EGFR* nor *KRAS* was detected in pericarcinomatous tissues, whereas *BRAF* and *RET* were detected. This result suggests that matched pericarcinomatous tissue might not act as normal control for detecting oncogenes. Remarkably, mutations in androgen receptor (*AR*), serine/threonine kinase 11 (*STK11*), *TP53*, neurofibromin 1 (*NF1*), *BRAF*, and neurogenic locus notch homolog protein 2 (*NOTCH2*) genes were detected in pericarcinomatous tissues, and these genes are associated with epithelial–mesenchymal transition (EMT). However, EMT is a complex process which is regulated by a multi-level molecular signalling pathway, instead of a single gene mutation. Some mutations of tumor-derived genes detected in pericarcinomatous tissues are related to EMT. However, their roles in EMT formation should be further explored and validated.

Although the present study showed several interesting findings, our conclusions may be affected by several limitations. First, the small cohort size was a limitation. Further study enrolling more patients is needed. Second, we did not measure and record the exact distance between the pericarcinomatous site and the primary tumor location. During the sample collection, the pericarcinomatous tissue was collected at the furthest distance (visible to the naked eyes) from the tumor in resected specimen (at least 5 cm from the tumor). As a result, it is not possible to evaluate whether those mutation-negative pericarcinomatous samples were further away from the primary tumor sites as compared with mutation-positive ones. Future studies are warranted to address this issue. Additionally, we used panel target capture sequencing instead of whole exome or genome sequencing for analyses, which might have resulted in some missing data, especially in passenger gene regions. However, we used a pan-cancer panel that included 1021 genes related to solid tumor carcinogenesis for sequencing analyses, and this panel showed a significant consistency with exome sequencing for detecting mutation number using the Pearson correlation analysis. Although we cannot rule out missing data entirely, panel sequencing may be a more applicable and cost-effective method for such analyses.

Tumor-derived mutations exist in pericarcinomatous tissues from patients with NSCLC, mostly enriched in trunk mutations and TSGs. Neither *EGFR* nor *KRAS* mutations were detected in pericarcinomatous tissues, whereas *BRAF* and *RET* were detected. It suggests that pericarcinomatous tissue should be neither recommended as filtering background for genomics analysis nor suitable for detecting druggable oncogenes of NSCLC.

## Additional files

**Additional file 1: Table S1.** List of genes in the pan-cancer 1021-gene panel listed according to their target regions. **Table S2.** Clinical characteristics of tumor-derived mutation detection in pericarcinomatous tissues from 32 enrolled patients with NSCLC.

**Additional file 2.** Additional Methods.

**Additional file 3: Figure S1.** The distributions of variant allele fraction and number of variant reads for patients with tumor-derived mutations detected in pericarcinomatous tissues. Box plot elements: center line, median; box limits, upper and lower quartiles; whiskers, 1.5 × interquartile range; points, outliers.

## Data Availability

The key raw data have been deposited into the Research Data Deposit (http://www.researchdata.org.cn), with the approval number of RDDB2019000525.

## Abbreviations

NGS: next-generation sequencing
NSCLC: non-small-cell lung cancer
EGFR: epidermal growth factor receptor
KRAS: kirsten rat sarcoma viral oncogene
TCGA: The Cancer Genome Atlas
TSG: tumor suppressor gene
BRAF: v-Raf murine sarcoma viral oncogene homolog B
EMT: epithelial–mesenchymal transition
