# Supplementary material for: Preferable background filtering for next-generation sequencing analysis in non-small cell lung cancer: pericarcinomatous tissues or peripheral blood lymphocytes?
Source: Cancer Commun (Lond). 2019 Jun 13;39:33. doi: 10.1186/s40880-019-0378-4 (PMC6563381; doi:10.1186/s40880-019-0378-4)
Supplement: Supplementary file 1 — Additional file 1: Table S1. List of genes in the pan-cancer 1021-gene panel listed according to their target regions. Table S2. Clinical characteristics of tumor-derived mutation detection in pericarcinomatous tissues from 32 enrolled patients with NSCLC. [file 40880_2019_378_MOESM1_ESM.docx]

**Table S1. List of genes in the pan-cancer 1021-gene panel listed according to their target regions.**

| **Coding sequence** | | | | | | | | |
| --- | --- | --- | --- | --- | --- | --- | --- | --- |
| *ABL1* | *BRD3* | *CDKN2B* | *FAT1* | *HDAC1* | *MCL1* | *NOTCH3* | *PTEN* | *SYK* |
| *ABL2* | *BRD4* | *CHEK1* | *FBXW7* | *HDAC4* | *MDM2* | *NOTCH4* | *PTPN11* | *TMPRSS2* |
| *AKT1* | *BTK* | *CHEK2* | *FCGR2A* | *HGF* | *MDM4* | *NRAS* | *RAF1* | *TOP1* |
| *AKT2* | *C11orf30* | *CRKL* | *FCGR2B* | *HRAS* | *MED12* | *NTRK1* | *RARA* | *TP53* |
| *AKT3* | *C1QA* | *CSF1R* | *FCGR3A* | *IDH1* | *MET* | *NTRK3* | *RB1* | *TSC1* |
| *ALK* | *C1S* | *CTNNB1* | *FGFR1* | *IDH2* | *MITF* | *PALB2* | *RET* | *TSC2* |
| *APC* | *CBL* | *DDR1* | *FGFR2* | *IGF1R* | *MLH1* | *PDGFRA* | *RHEB* | *VEGFA* |
| *AR* | *CCND1* | *DDR2* | *FGFR3* | *IL7R* | *MLH3* | *PDGFRB* | *RHOA* | *VHL* |
| *ARAF* | *CCND2* | *DNMT3A* | *FGFR4* | *INPP4B* | *MPL* | *PDK1* | *RICTOR* | *XPO1* |
| *ATM* | *CCND3* | *EGFR* | *FLCN* | *IRS2* | *MS4A1* | *PIK3CA* | *RNF43* | *XRCC1* |
| *ATR* | *CCNE1* | *EPHA2* | *FLT1* | *JAK1* | *MSH2* | *PIK3CB* | *ROCK1* |  |
| *AURKA* | *CD274* | *EPHA3* | *FLT3* | *JAK2* | *MSH3* | *PIK3R1* | *ROS1* |  |
| *AURKB* | *CDH1* | *EPHA5* | *FLT4* | *JAK3* | *MSH6* | *PIK3R2* | *RPS6KB1* |  |
| *AXL* | *CDK13* | *ERBB2* | *FOXA1* | *KDR* | *MTOR* | *PMS1* | *SMARCA4* |  |
| *BAP1* | *CDK4* | *ERBB3* | *FOXL2* | *KIT* | *MYC* | *PMS2* | *SMARCB1* |  |
| *BCL2* | *CDK6* | *ERBB4* | *GAB2* | *KRAS* | *MYD88* | *PRKAA1* | *SMO* |  |
| *BRAF* | *CDK8* | *ERCC1* | *GATA3* | *MAP2K1* | *NF1* | *PSMB1* | *SRC* |  |
| *BRCA1* | *CDKN1A* | *ERG* | *GNA11* | *MAP2K2* | *NF2* | *PSMB5* | *STAT1* |  |
| *BRCA2* | *CDKN1B* | *ESR1* | *GNAQ* | *MAPK1* | *NOTCH1* | *PTCH1* | *STAT3* |  |
| *BRD2* | *CDKN2A* | *EZH2* | *GNAS* | *MAPK3* | *NOTCH2* | *PTCH2* | *STK11* |  |
| **Hot exons** | | | | | | | | |
| *ABCA10* | *CAPRIN1* | *DMXL1* | *GLYR1* | *LMAN1L* | *NXF5* | *RALBP1* | *STAG2* | *UNC13A* |
| *ABCA8* | *CARS* | *DMXL2* | *GMDS* | *LMBR1L* | *OBP2A* | *RAPGEF2* | *STAT4* | *UNC13D* |
| *ABCB7* | *CARS2* | *DNAH10* | *GNPTAB* | *LPCAT4* | *OBP2B* | *RARB* | *STAT6* | *UNC5D* |
| *ABCC8* | *CASC4* | *DNAH5* | *GOLGA4* | *LPHN3* | *OCA2* | *RASEF* | *STK11IP* | *USP12* |
| *ABCF2* | *CASP8* | *DNAH9* | *GPAT2* | *LRBA* | *ODZ3* | *RBM6* | *STK31* | *USP34* |
| *ACE* | *CASP8AP2* | *DNAJC11* | *GPATCH2* | *LRP1B* | *OR2T4* | *RBMX* | *STX3* | *USP39* |
| *ACER2* | *CASQ2* | *DNAJC9* | *GPR114* | *LRP2* | *OR4A15* | *RCC1* | *SULT1A4* | *USP45* |
| *ACOT11* | *CATSPER2* | *DNTTIP1* | *GPR125* | *LRP4* | *OR4C6* | *REC8* | *SUPT5H* | *USP48* |
| *ACPP* | *CBFB* | *DOCK11* | *GPR133* | *LRRC16B* | *OR5L2* | *REG1B* | *SUPT6H* | *VAV1* |
| *ACSL1* | *CBX4* | *DOCK3* | *GPR144* | *LRRC2* | *OR6F1* | *RELN* | *SYCP2L* | *VEZF1* |
| *ACSM5* | *CCDC155* | *DOT1L* | *GPS2* | *LRRC7* | *OSBPL10* | *RERE* | *SYNE1* | *VILL* |
| *ACSS3* | *CCDC159* | *DPP10* | *GRIA3* | *LRRC72* | *OTOA* | *RFWD2* | *SYNE2* | *VIT* |
| *ACTL6B* | *CCDC17* | *DPP4* | *GRIK2* | *LRRD1* | *OTOGL* | *RFX3* | *SYNJ2* | *VPS13A* |
| *ADAM23* | *CCT3* | *DRGX* | *GUCY1A3* | *LRRFIP2* | *OVCH1* | *RNF215* | *TAF1B* | *VPS33B* |
| *ADAM33* | *CCT6B* | *DUOX1* | *GUCY2C* | *LRSAM1* | *P4HB* | *RNF219* | *TAF6* | *VSIG4* |
| *ADAMTS12* | *CD1E* | *DYSF* | *GYLTL1B* | *LTBP1* | *PABPC4* | *RPL22* | *TARBP1* | *WAS* |
| *ADAMTS16* | *CD300LF* | *DZANK1* | *HAAO* | *LUC7L2* | *PACS2* | *RPL36A* | *TBC1D1* | *WASL* |
| *ADAMTS19* | *CD5L* | *ECHDC1* | *HAP1* | *LUZP4* | *PAEP* | *RPS5* | *TBC1D21* | *WDR44* |
| *ADAMTS20* | *CD9* | *EDN1* | *HAUS5* | *MAEL* | *PAGE1* | *RPS6KA1* | *TBC1D3* | *WDR52* |
| *ADAMTS5* | *CD97* | *EEF1A1* | *HAUS6* | *MAGI1* | *PARK2* | *RPTOR* | *TBC1D5* | *WDR62* |
| *ADAMTSL1* | *CD99* | *EFCAB5* | *HCN1* | *MAN2A1* | *PARP4* | *RPUSD4* | *TBL1X* | *WDR66* |
| *ADD2* | *CDH18* | *EFCAB6* | *HDAC6* | *MAP2* | *PCK2* | *RREB1* | *TBP* | *WDR72* |
| *AGMAT* | *CDH24* | *EFCAB7* | *HEATR7B2* | *MAP2K4* | *PCLO* | *RRP7A* | *TBX15* | *WDTC1* |
| *AGTPBP1* | *CDH26* | *EFHA2* | *HECTD4* | *MAP3K1* | *PCNT* | *RUNDC3A* | *TBX22* | *WLS* |
| *AHCTF1* | *CDK11A* | *EFNA5* | *HECW1* | *MAP4K1* | *PCNXL2* | *RUNX1* | *TBX3* | *WSCD2* |
| *AK5* | *CDK12* | *EIF1AX* | *HECW2* | *MAPKAPK3* | *PCSK5* | *RYR2* | *TCF20* | *WWP2* |
| *AKR1B10* | *CDK14* | *EIF2B5* | *HID1* | *MAPRE3* | *PCYT1A* | *RYR3* | *TCF4* | *XBP1* |
| *AKR1C1* | *CDK18* | *EIF2C2* | *HIST1H3B* | *MAST1* | *PDCD6* | *SAFB2* | *TCP10* | *XPO4* |
| *ALDH1A3* | *CDK19* | *EIF3E* | *HLA-DRB1* | *MBIP* | *PDE1C* | *SAG* | *TCP11* | *XPO5* |
| *ALDH2* | *CDS1* | *EIF3I* | *HLA-DRB5* | *MBTPS2* | *PDE2A* | *SAGE1* | *TEK* | *ZAP70* |
| *ALG5* | *CEACAM20* | *EIF4ENIF1* | *HMCN1* | *MCF2L2* | *PDE4DIP* | *SAMD8* | *TERT* | *ZBTB8OS* |
| *ALX4* | *CECR2* | *EIF4H* | *HMHA1* | *MCOLN2* | *PDIA5* | *SCN10A* | *TESC* | *ZC3H13* |
| *AMOT* | *CELA2B* | *ELAVL3* | *HNF4A* | *MDGA2* | *PDILT* | *SCN3A* | *TEX35* | *ZC3H7B* |
| *ANK2* | *CGN* | *ELL3* | *HOMER2* | *MDN1* | *PDRG1* | *SCN7A* | *TFDP1* | *ZDHHC11* |
| *ANKRD13D* | *CHD3* | *EMID2* | *HPS3* | *MED23* | *PEX6* | *SCN9A* | *TGDS* | *ZFC3H1* |
| *ANKRD20A4* | *CHD4* | *ENPP2* | *HPS4* | *MEFV* | *PGAP1* | *SDK2* | *TGM2* | *ZFR* |
| *ANKRD27* | *CHD6* | *ENTPD6* | *HSPA12B* | *METTL14* | *PHACTR3* | *SEC14L4* | *TGM5* | *ZMYM4* |
| *ANKRD28* | *CHI3L1* | *EPB41L2* | *HSPD1* | *METTL5* | *PHF20L1* | *SEC24B* | *THBS2* | *ZNF143* |
| *ANKRD30A* | *CISD3* | *EPB41L4B* | *HYDIN* | *MGAM* | *PHYH* | *SEH1L* | *THEM5* | *ZNF350* |
| *ANKRD30B* | *CLCN7* | *EPHB1* | *IBSP* | *MICALL1* | *PI4KB* | *SELP* | *THOC1* | *ZNF385A* |
| *ANKRD36B* | *CLEC16A* | *EPS8L3* | *IFT172* | *MID1* | *PIP4K2C* | *SEMA6A* | *THSD7A* | *ZNF414* |
| *ANO2* | *CLINT1* | *ESD* | *IGSF9* | *MIER2* | *PIP5K1C* | *SEPT12.* | *THSD7B* | *ZNF512B* |
| *AP1B1* | *CNGB3* | *ETNK2* | *IKBKAP* | *MLL3* | *PIWIL1* | *SERPINA7* | *TIMD4* | *ZNF541* |
| *AP1G2* | *CNKSR2* | *ETV6* | *IKBKE* | *MLPH* | *PKD1L2* | *SETD1B* | *TIMM44* | *ZNF563* |
| *AP3B1* | *CNOT3* | *EXOC4* | *IL11RA* | *MORC1* | *PKHD1* | *SETD2* | *TIMP3* | *ZNF614* |
| *APAF1* | *CNOT4* | *EXOC5* | *IL13RA2* | *MORN1* | *PKLR* | *SF1* | *TJP3* | *ZNF687* |
| *APLP2* | *CNTN1* | *EXOC6* | *IL1RAPL1* | *MRPL1* | *PLAC8* | *SF3B1* | *TLE1* | *ZNF705B* |
| *APMAP* | *CNTN4* | *EXOC7* | *IL27RA* | *MRPL24* | *PLCB4* | *SF3B14* | *TLL1* | *ZNF705G* |
| *APPL2* | *CNTN5* | *EXTL3* | *IMPG1* | *MRPS18B* | *PLCZ1* | *SF3B3* | *TMC2* | *ZNF711* |
| *AQP12A* | *CNTNAP3B* | *EYA4* | *INHBA* | *MSI1* | *PLEC* | *SGCZ* | *TMED8* | *ZNF804B* |
| *ARFGAP1* | *CNTNAP5* | *F8* | *INPP5J* | *MTA2* | *PLK2* | *SGIP1* | *TMEM104* | *ZSWIM8* |
| *ARFRP1* | *COASY* | *F9* | *IQCA1* | *MTM1* | *PLOD3* | *SGK1* | *TMEM120B* |  |
| *ARHGAP35* | *COL14A1* | *FAH* | *ITFG2* | *MTR* | *PLXNA1* | *SGPL1* | *TMEM132D* |  |
| *ARHGAP40* | *COL16A1* | *FAM114A2* | *ITGA8* | *MTTP* | *POLDIP2* | *SH2D3A* | *TMEM145* |  |
| *ARHGEF1* | *COL19A1* | *FAM131B* | *ITGA9* | *MUC5B* | *POLE* | *SH3BGR* | *TMEM247* |  |
| *ARHGEF7* | *COL1A1* | *FAM135B* | *ITIH1* | *MUS81* | *POLR2J* | *SH3PXD2A* | *TMEM80* |  |
| *ARNTL* | *COL25A1* | *FAM13C* | *ITLN2* | *MYB* | *POLR3B* | *SHISA4* | *TMEM87A* |  |
| *ARPC4-TTLL3* | *COL4A5* | *FAM157B* | *ITM2A* | *MYBPC2* | *POLR3GL* | *SI* | *TMTC4* |  |
| *ASH2L* | *COL4A6* | *FAM177B* | *ITPKB* | *MYCBP2* | *POLRMT* | *SIDT2* | *TMX3* |  |
| *ASTN1* | *COL5A1* | *FAM21A* | *ITPR1* | *MYH15* | *POM121L12* | *SIK3* | *TNFAIP6* |  |
| *ASXL2* | *COL5A2* | *FAM3A* | *KCNAB2* | *MYH2* | *POTEG* | *SIM1* | *TNFSF4* |  |
| *ATAD2B* | *COL5A3* | *FAM49A* | *KCNH6* | *MYH4* | *PPA1* | *SIM2* | *TNN* |  |
| *ATG9B* | *COL6A5* | *FAM49B* | *KCNQ2* | *MYH8* | *PPDPF* | *SLC13A3* | *TNNT1* |  |
| *ATP10B* | *COL6A6* | *FAM5C* | *KDM4A* | *MYH9* | *PPEF1* | *SLC17A6* | *TNR* |  |
| *ATP10D* | *COL9A1* | *FAM86B1* | *KDM6A* | *MYL5* | *PPFIBP2* | *SLC17A8* | *TNS3* |  |
| *ATP12A* | *COPA* | *FAN1* | *KEAP1* | *MYL6* | *PPIL2* | *SLC25A1* | *TP53BP1* |  |
| *ATP2C1* | *COPG1* | *FANCC* | *KIAA0195* | *MYLK2* | *PPP1R17* | *SLC25A30* | *TPCN1* |  |
| *ATP6V0A2* | *CPA1* | *FASTK* | *KIAA0226* | *MYO3A* | *PPP4R4* | *SLC26A3* | *TPH2* |  |
| *ATP8B2* | *CPSF3* | *FATE1* | *KIAA0319* | *MYOM1* | *PQBP1* | *SLC2A2* | *TPMT* |  |
| *ATXN2* | *CPSF6* | *FBN2* | *KIAA0922* | *NACAD* | *PREB* | *SLC30A5* | *TPTE* |  |
| *ATXN7L2* | *CRTAM* | *FDCSP* | *KIAA1191* | *NARF* | *PREX2* | *SLC35B2* | *TRIM33* |  |
| *BAX* | *CRTAP* | *FLNC* | *KIAA1199* | *NAT10* | *PRKACA* | *SLC35B4* | *TRIM51* |  |
| *BBS9* | *CRYBG3* | *FLOT2* | *KIAA1211L* | *NAV3* | *PRKAG3* | *SLC38A4* | *TRIM58* |  |
| *BCAS1* | *CSMD1* | *FLT3LG* | *KIF13A* | *NBPF1* | *PRKCD* | *SLC38A5* | *TRIML1* |  |
| *BCAS2* | *CSMD3* | *FMN2* | *KIF1B* | *NBPF10* | *PRKDC* | *SLC43A1* | *TRIO* |  |
| *BCL2L11* | *CSN3* | *FMNL3* | *KIF26B* | *NCF2* | *PRKX* | *SLC45A1* | *TRIP11* |  |
| *BCR* | *CSNK1E* | *FNDC4* | *KIF5B* | *NCKAP1* | *PRRX1* | *SLC4A10* | *TRMT112* |  |
| *BLOC1S1* | *CSPP1* | *FNIP2* | *KIFAP3* | *NCOR1* | *PRSS1* | *SLC4A4* | *TRPC5* |  |
| *BMPR1B* | *CTCF* | *FOLH1* | *KIFC1* | *NCOR2* | *PRUNE* | *SLC5A1* | *TRUB1* |  |
| *BRF1* | *CTIF* | *FOXJ2* | *KIR2DL3* | *NEK5* | *PSG2* | *SLC6A5* | *TSGA10* |  |
| *BRSK2* | *CTNNA2* | *FRG1* | *KIR3DL3* | *NELL1* | *PSG5* | *SLC8A1* | *TSKS* |  |
| *BRWD3* | *CTSF* | *FRG2B* | *KLHL1* | *NFE2L2* | *PSIP1* | *SLCO1B7* | *TSPAN12* |  |
| *BSG* | *CYP2A13* | *FRMD4A* | *KLHL14* | *NIPBL* | *PSMC4* | *SLCO5A1* | *TSR2* |  |
| *BTNL3* | *CYP3A4* | *FRMPD2* | *KLK1* | *NLGN3* | *PSMC6* | *SMTN* | *TTF2* |  |
| *BTRC* | *CYP4A11* | *FRMPD4* | *KMT2B* | *NLRC3* | *PSTPIP1* | *SNTG1* | *TTN* |  |
| *C12orf5* | *CYTH4* | *FSD2* | *KMT2C* | *NLRP4* | *PTBP3* | *SORCS3* | *TUBA3C* |  |
| *C19orf38* | *DCLK2* | *FSHR* | *KRT2* | *NMI* | *PTCD3* | *SPAG16* | *TUBGCP4* |  |
| *C1orf112* | *DCST1* | *FUBP1* | *KRT9* | *NOP2* | *PTGES3L-AARSD1* | *SPATA13* | *TUBGCP5* |  |
| *C1orf35* | *DDB1* | *FUNDC1* | *KRTAP5-5* | *NOS1* | *PTGS2* | *SPG20* | *TYK2* |  |
| *C20orf112* | *DDX24* | *GAB3* | *KTN1* | *NOS2* | *PTPLAD1* | *SPINT1* | *TYRP1* |  |
| *C2orf47* | *DDX3X* | *GABRD* | *L3MBTL1* | *NRXN1* | *PTPN13* | *SPPL2A* | *U2AF1* |  |
| *C2orf62* | *DEPDC4* | *GAD2* | *LARP1* | *NRXN2* | *PTPRA* | *SPPL3* | *U2AF2* |  |
| *C7orf53* | *DGKK* | *GALNT13* | *LCN10* | *NT5C3L* | *PTPRD* | *SPRED1* | *UBASH3A* |  |
| *C9orf114* | *DHCR24* | *GALNT14* | *LCT* | *NTM* | *PTPRM* | *SPTA1* | *UBE2Q1* |  |
| *C9orf43* | *DHDDS* | *GFRAL* | *LCTL* | *NUDCD2* | *PYHIN1* | *SRRT* | *UBE4B* |  |
| *CACNA1A* | *DHX9* | *GIGYF1* | *LETM1* | *NUP205* | *QRICH2* | *SSBP3* | *UCHL3* |  |
| *CACNA1D* | *DIAPH1* | *GINS4* | *LGALS13* | *NUP210* | *RAB1B* | *SSH2* | *UCK2* |  |
| *CACNA1E* | *DKC1* | *GIPR* | *LILRB3* | *NUTM1* | *RAB3GAP2* | *SSPO* | *UGT8* |  |
| *CADM2* | *DLST* | *GKN2* | *LILRB4* | *NWD1* | *RAB6A* | *ST18* | *ULK3* |  |
| *CAMKK1* | *DMD* | *GLB1L3* | *LIPN* | *NXF1* | *RAC2* | *ST6GALNAC1* | *UMOD* |  |

**Table S2. Clinical characteristics of tumor-derived mutation detection in pericarcinomatous tissues from 32 enrolled patients with NSCLC.**

| **Characteristic** | **Total (cases)** | **Mutation in pericarcinomatous tissues [cases (%)]** | ***P* value^*^** |
| --- | --- | --- | --- |
| **Age (years)** |  |  |  |
| 45-59 | 18 | 2 (11.1) | Ref. |
| 60-69 | 11 | 2 (18.2) | 0.622 |
| 70-79 | 3 | 2 (66.7) | 0.080 |
| **Gender** |  |  |  |
| Male | 25 | 5 (20.0) | Ref. |
| Female | 7 | 1 (14.3) | 1.000 |
| **NSCLC subtype** |  |  |  |
| *EGFR-*mutant adenocarcinoma | 9 | 2 (22.2) | Ref. |
| *KRAS-*mutant adenocarcinoma | 6 | 1 (16.7) | 1.000 |
| *EGFR*&*KRAS* wild-type adenocarcinoma | 11 | 2 (18.2) | 1.000 |
| Squamous cell carcinoma | 5 | 1 (20.0) | 1.000 |
| Lymphoepithelioma-like carcinoma | 1 | 0 (0) | 1.000 |
| **TNM stage** |  |  |  |
| I | 12 | 1 (8.3) | Ref. |
| II | 8 | 4 (50.0) | 0.109 |
| III | 10 | 1 (10.0) | 1.000 |
| IV | 2^#^ | 0 (0) | 1.000 |
| **Smoking status** |  |  |  |
| Non-smoking | 11 | 1 (9.1) | Ref. |
| Smoking | 21 | 5 (23.8) | 0.637 |

^*^ Fisher’s exact test was employed to compare the detection rate of tumor-derived mutations in pericarcinomatous tissues among different subsets stratified by clinical characteristics.

^#^Both patients had solitary brain metastasis and underwent brain radical surgery.

Abbreviations: NSCLC: non-small cell lung cancer; *EGFR*: epidermal growth factor receptor; *KRAS*: kirsten rat sarcomaviral oncogene.
