# Supplementary material for: Preferable background filtering for next-generation sequencing analysis in non-small cell lung cancer: pericarcinomatous tissues or peripheral blood lymphocytes?
Source: Cancer Commun (Lond). 2019 Jun 13;39:33. doi: 10.1186/s40880-019-0378-4 (PMC6563381; doi:10.1186/s40880-019-0378-4)
Supplement: Supplementary file 2 — Additional file 2. Additional Methods. [file 40880_2019_378_MOESM2_ESM.docx]

**Additional Methods S1**

**Sample collection**

All enrolled patients underwent pulmonary surgery at the Sun Yat-sen University Cancer Center (Guangzhou, Guangdong, China) between June 2016 and March 2017. Tumor samples were assessed by two pathologists from Department of Pathology of Sun Yat-sen University Cancer Center to confirm the diagnosis of non-small cell lung cancer (NSCLC). We used the version 7 TNM staging system released by American Joint Committee of Cancer (AJCC) in this study. We collected multi-region tumor tissues, pericarcinomatous tissues, matched peripheral blood, and clinical information from each patient. During the sample collection, we selected multi-region tumor tissues in different quadrants as shown in **Figure 1**, while the pericarcinomatous tissue was collected at the furthest distance (visible to the naked eyes) from the tumor in resected specimen (at least 5cm from the tumor). There was no contamination of pericarcinomatous tissue by tumor cells. Tissues from all regions were collected with sterile blades. All patients provided written informed consent before surgery. This study was approved by the Institutional Review Board (IRB) of Sun Yat-sen University Cancer Center (IRB number B2017-067-01).

**DNA extraction and sequencing**

Peripheral blood was collected in EDTA Vacutainer tubes (BD Diagnostics, Franklin Lakes, NJ, USA) and processed to separate peripheral blood lymphocytes (PBLs) as previously described [1]. DNA was extracted from PBLs and tissue samples using the DNeasy Blood & Tissue Kit (Qiagen, Hilden, Germany) according to the manufacturer’s instructions. Sequencing libraries were constructed with the KAPA DNA Library Preparation Kit (KapaBiosystems, Wilmington, MA, USA) according to the manufacturer’s protocol. Libraries were hybridized to custom-designed biotinylated oligonucleotide probes (Integrated DNA Technologies, Iowa, IA, USA). Capture probes were designed to cover coding sequences or hot exons of 1021 genes frequently mutated in solid tumors (Supplementary Table S1). DNA sequencing was performed using the HiSeq 3000 Sequencing System (Illumina, San Diego, CA, USA) with 200-bp paired-end reads.

**Mutation analysis**

Targeted capture sequencing was performed in 181 samples of multi-region tumor tissues and 32 samples of matched pericarcinomatous tissues from 32 patients with resectable NSCLC using a pan-cancer 1021-gene panel [2]. The mean effective depth of sequencing was 810× (ranged from 195× to 1302×). Tumor-derived mutations were identified. Then, we rescued these mutations in matched pericarcinomatous tissues. Single nucleotide variants (SNVs) were called using MuTect (version 1.1.4) [3] and NChot, a software developed in-house to review hotspot variants. Small insertions and deletions were called by GATK (website: https://software.broadinstitute.org/gatk/) [4]. If a somatic genetic alteration was shared by all tumor tissue regions, it was defined as trunk mutation. Otherwise, it was called branch mutation. Mutations of tumor-derived genes detected in pericarcinomatous tissues need to meet following criteria: i) variant allele fraction ≥ 1%, or ii) variant allele read number ≥ 5, but not just present weak signal. Thus, these mutations were identified in tumors and present reliable mutant reads in pericarcinomatous tissues.

**Statistical analysis**

Chi-square test was employed to compare the proportions of trunk mutations and branch mutations between tumor tissues and pericarcinomatous tissues. Fisher’s exact test was used to compare the detection rate of tumor-derived gene mutations in pericarcinomatous tissues among different subsets stratified by clinical characteristics. All statistical analyses were performed with SPSS (v.21.0; STATA, College Station, TX, USA) or GraphPad Prism software (v. 6.0; GraphPad Software, La Jolla, CA, USA). Statistical significance was defined as a two-sided *P*<0.05.

**References**

1 Nong J, Gong Y, Guan Y, et al. Circulating tumor DNA analysis depicts subclonal architecture and genomic evolution of small cell lung cancer. Nature communications 2018;9:3114.

2 Zhang Y, Chang L, Yang Y, et al. Intratumor heterogeneity comparison among different subtypes of non-small-cell lung cancer through multi-region tissue and matched ctdna sequencing. Molecular cancer 2019;18:7.

3 Cibulskis K, Lawrence MS, Carter SL, et al. Sensitive detection of somatic point mutations in impure and heterogeneous cancer samples. Nature biotechnology 2013;31:213-219.

4 Hu ZY, Xie N, Tian C, et al. Identifying circulating tumor DNA mutation profiles in metastatic breast cancer patients with multiline resistance. EBioMedicine 2018;32:111-118.
